# Comprehensive Understanding of the Effect of TGO Growth Modes on Thermal Barrier Coating Failure Based on a Simulation

**DOI:** 10.3390/ma17010180

**Published:** 2023-12-28

**Authors:** Da Qiao, Jixin Man, Wengao Yan, Beirao Xue, Xiangde Bian, Wu Zeng

**Affiliations:** 1 Advanced Gas Turbine Laboratory, Institute of Engineering Thermophysics, Chinese Academy of Sciences, Beijing 100190, China; qiaoda@iet.cn (D.Q.); manjixin@iet.cn (J.M.); yanwengao@iet.cn (W.Y.); xuebeirao@iet.cn (B.X.); bianxiangde@iet.cn (X.B.); 2 University of Chinese Academy of Sciences, Beijing 100049, China; 3 National Key Laboratory of Science and Technology on Advanced Light-Duty Gas-Turbine, Beijing 100190, China; 4 Key Laboratory of Advanced Energy and Power, Institute of Engineering Thermophysics, Chinese Academy of Sciences, Beijing 100190, China

**Keywords:** thermal barrier coatings (TBCs), TGO growth, cracking, failure behavior

## Abstract

The growth stress induced by thermally grown oxide (TGO) is one of the main reasons for the failure of thermal barrier coatings (TBCs). In this study, the failure behavior of TBCs was examined based on different growth modes of TGO. A TBC thermo-mechanical model with a simplified sinusoidal interface morphology was established by the secondary development of a numerical simulation. The plasticity and creep behavior of materials were considered. Based on the subroutine development, the non-uniform growth of the TGO layer was realized. Cohesive elements were also applied to the TC/TGO interface. The stress distribution and evolution at the TC/TGO interface were investigated. Then, the cracking behavior near the interface was studied. The results show that lateral growth causes the off-valley site to replace the previous off-peak site as a vulnerable site. The non-uniform growth accelerates damage in the off-valley site, which leads to a change in the failure behavior. These results will provide significant guidance for understanding the TBC failure and the development of advanced TBCs.

## 1. Introduction

**Figure 1:**
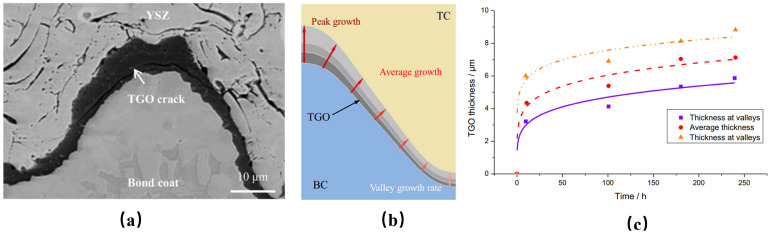
Non-uniform growth of TGO: (**a**) cross-sectional observation [19,20]; (**b**) schematic diagram [21]; and (**c**) variation of different areas with heating time [21].

**Figure 2:**
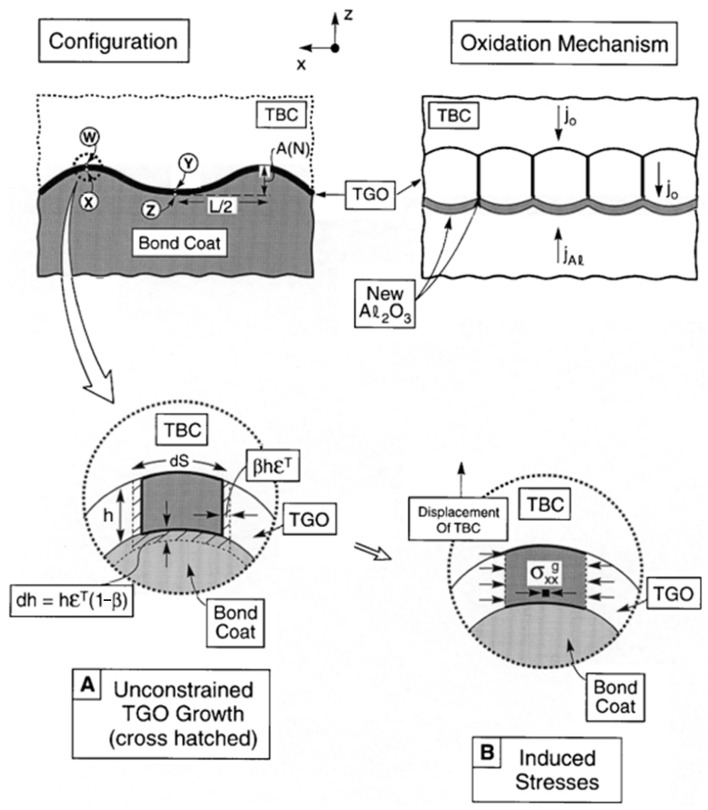
TGO growth schematic [22].

Thermal barrier coatings (TBCs) have been widely used as a more effective way to insulate heat to improve the efficiency of gas turbines. Typical TBCs generally consist of two layers: a ceramic topcoat (TC), which acts as a thermal insulator, and a bond coat (BC), which acts as an antioxidant layer [1,2,3]. During the service process, the TBCs will fail mainly in the form of coating peeling. Coatings prepared by atmospheric plasma spraying (APS) have a porous microstructure with low strain tolerance, and the internal holes play a role in promoting the emergence and propagation of cracks. When the interfacial cracks extend to a certain extent, the coating peels off. Many factors can result in the change in stress distributions: the plastic deformation of each layer [4,5]; creep at high temperatures [6,7,8]; ceramic sintering [9,10,11]; CMAS corrosion; and the impact of foreign objects [12,13,14]. The growth stress caused by thermally grown oxide (TGO) is the main cause of coating failure [15,16,17,18]. The appearance of the TGO layer occurs because ceramics barely hinder oxygen diffusion. At high temperatures, oxygen passes through the ceramic layer and chemically reacts with Al elements from the bonding coat, which will cause significant growth stresses on the TC/BC. At the same time, due to the large difference in physical parameters from the TC/BC, a large thermal mismatch stress can be also introduced. The growth of TGO is a chemical reaction resulting from the diffusion of elements. The thickness of TGO varies from area to area as a result of the diffusion process. The growth rate at the peak areas will be higher than in the valley areas, as shown in Figure 1. TGO is a new substance generated by a chemical reaction. The schematic diagram of the growth is shown in Figure 2. It is generally believed that it grows in two directions: the thickening direction and the lateral direction.

A large number of simulation studies have been carried out to characterize the effect of TGO. Early studies only considered the effect of TGO thickness on stress. Yu et al. [23] investigated the effect of interface roughness and TGO thickness on coating stress and found that the higher the TGO thickness, the higher the residual stress. Afterward, Chen et al. [24] investigated the effect of the non-uniform thickness of TGO and found that coatings with non-uniform thicknesses were more prone to failure. However, none of these models considered growth strain in the TGO thickening and lateral direction. However, some studies considering the growth in these two directions began to appear. Song et al. [25] discussed the stress evolution and cracking behavior of coatings during thermal cycling based on the consideration of the growth strain in only the thickening direction. Similarly, Wei et al. [14] also used the VCCT method to capture and study the crack extension behavior during thermal cycling without considering lateral growth. For lateral growth strain, the lateral kinetics of oxidation are still unclear due to a lack of experimental data. The lateral growth of TGO is generally performed directly by parametric analysis. Wei et al. [26] investigated the effect of TGO lateral growth on coating stress as well as on the failure behavior during thermal cycling and demonstrated that lateral growth leads to the coating being more susceptible to spallation failure. Clarke et al. [27] proposed that the lateral growth strain of TGO is linearly related to the growth strain in the thickening direction and conducted a related study.

In previous studies, TGO growth was generally considered to be uniform. However, in the experimental observation, it was found that, due to the influence of interfacial morphology and elemental diffusion, the growth strain of TGO during the growth process generally decreased from the peak to the valley, with thicker TGO at the peak and thinner TGO at the valley [28]. Liu et al. [29] considered multiple growth modes of TGO and investigated the coating stress evolution and cracking behavior during thermal cycling but did not consider the effect of lateral growth. Wei et al. [11,30] investigated the effect of TGO growth modes on the crack extension and merging mechanism. However, there was no systematic analysis of the TGO growth mode to study the effects of different growth modes on the stress distribution of the coating, the evolution of stress in different parts of the coating, or the effects of different growth modes on the cracking behavior.

In this paper, a sinusoidal interface based on the finite element software ABAQUS2020 was established, and the plasticity and creep behavior of materials were considered. Different TGO growth modes were realized using subroutine development. Uniform/non-uniform TGO growth with, and without, lateral growth were studied to examine the stress evolution. The cohesive element at the interface was introduced to systematically analyze the TBC failure behavior induced by TGO. The aim of the study was to obtain a more comprehensive understanding of the failure mechanisms of different TGO growth modes at the TC/TGO interface.

## 2. Numerical Model Development

### 2.1. Geometry and Meshing

**Figure 3:**
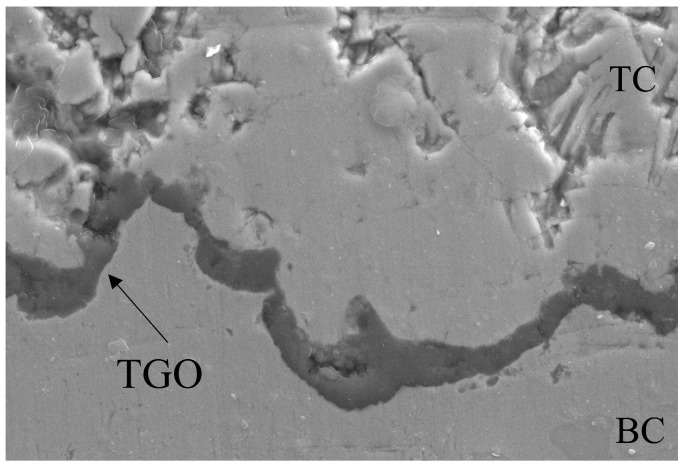
Characterization of typical coating microstructure.

The interface of the TC/BC is extremely complex and presents rough characteristics (see Figure 3). In this study, an ideal sinusoidal curve is used to fit the interface in a simplified approach, which can be represented using $y\left(x\right)=A_{0}\cdot \sin\left(2\pi x/\lambda \right)$. The wavelength *λ* and amplitude *A*_0_ are extracted from the representative volume element (RVE) region and satisfy $A_{0}/\lambda =0.5$ and λ=0.06mm, which has been widely used by scholars. Furthermore, due to the symmetry and periodicity, the model only contains a half of cycle.

**Figure 4:**
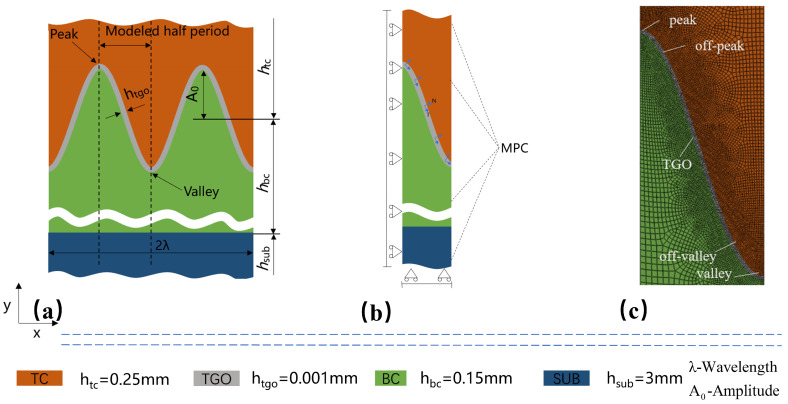
Adopted finite element model: (**a**) geometric model; (**b**) boundary conditions; and (**c**) mesh details and different parts of the interface definition.

The geometric model used for this simulation is shown in Figure 4. The TBC system consists of four layers: a topcoat (TC) with a thickness of 0.25 mm; a bond coat (BC) with a thickness of 0.15 mm; thermally grown oxide (TGO) with an initial thickness of 0.001 mm; and a substrate (SUB) with a thickness of 3 mm. In this simulation, the TGO is considered to be uniformly dense *a*-Al_2_O_3_, which has been adopted by many authors [30,31]. In addition, the low content of spinel oxides in TGO during the early stages of oxidation has a complex effect on the failure of TBCs and has been temporarily neglected [32].

### 2.2. Material Property

The TBC system studied in this study is composed of a DZ411 substrate, a NiCoCrAlY bond coat and an yttria partially stabilized zirconia (ZrO_2_-8 wt% Y_2_O_3_) topcoat. The thickness of each layer is 3 mm, 0.15 mm, and 0.25 mm. The initial thickness of the TGO layer between the TB and BC is assumed as 0.001 mm (see Figure 4). All materials involved in the simulations are considered to be homogeneous and isotropic. The thermo-physical properties, such as the coefficient of thermal expansion, modulus of elasticity, Poisson’s ratio, etc., vary with temperature and are demonstrated in Table 1 [33].

Due to the brittle nature of the topcoat (TC) and the very low creep rate at high temperatures, it is considered that the topcoat (TC) only exhibits elastic behavior. The yield strength [34,35] is temperature-dependent and decreases dramatically at high temperatures. It is very necessary to take them into account when performing the simulations; otherwise, the stress distributions and values that do not correspond to reality will be obtained. The yield strength for each layer is illustrated in Table 2.

The creep behavior of the BC is particularly evident when the coating temperature is greater than 600 °C. The creep of the TC as well as that of the thermally grown oxide (TGO) occurs only at high temperatures, and the time-dependent creep behavior is expressed by the following creep Norton equation:

(1) $${\dot{\varepsilon }}_{\mathrm{cr}}=B\sigma^{n}$$

where ${\dot{\varepsilon }}_{\mathrm{cr}}$ and σ are the strain rate and stress, respectively. B and n are the temperature-dependent parameters fitted experimentally, as shown in Table 3 [36]. The implementation is done through the user subroutine “CREEP” in Abaqus/standard.

### 2.3. Boundary Condition and Thermal Loading History

Due to the periodicity of the selected model, a symmetric constraint along the horizontal direction is imposed for the left side, and the right side is constrained using a multipoint constraint (MPC) to realize the periodicity of the model. This MPC constraint allows the nodes on the right side to move with the same displacement in the horizontal direction while allowing for free movement along the vertical direction. In addition, to avoid rigid body displacement in the model, a constraint along the vertical direction is imposed for the bottom boundary, and the boundary conditions are shown in Figure 4b.

**Figure 5:**
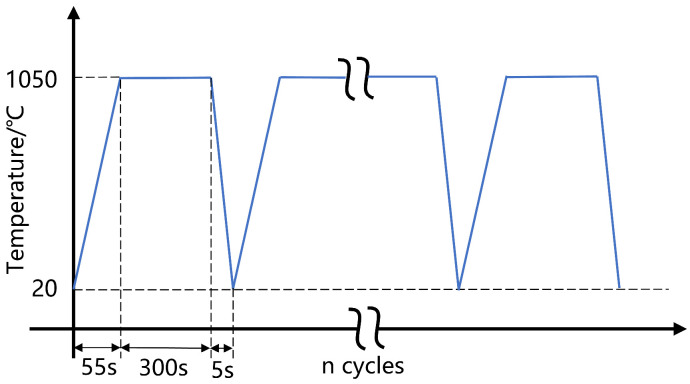
History of thermal cycles experienced.

The user subroutine DLOAD is employed to apply a uniform temperature field across the model without heat transfer during temperature changes. Creep at high temperatures allows for the release of stresses in the TBC. For the initial unstressed temperature set to 1000 °C, the thermal history experienced by the whole model is shown in Figure 5. For a single cycle, it was increased from 25 °C to 1050 °C after 55 s, followed by a holding phase of 300 s, and then cooled down to 25 °C after 5 s. A total of 20 cycles is examined.

### 2.4. Dynamic TGO Growth Modeling

Since the TC layer barely hinders the diffusion of oxygen ions, the oxygen diffuses through the TC and the elements, such as Al, and Cr from the bond coat (BC), to generate TGO through a chemical reaction under high-temperature conditions (>900 °C). It is mainly characterized by thickness growth perpendicular to the interface and lateral growth parallel to the interface, and the ratio of the growth rate of lateral growth to thickness growth is generally 0.1 [37,38]. The growth of TGO will introduce greater growth stresses to the coating interface. The simulation of TGO growth in ABAQUS was performed through the CREEP user subroutine. In this simulation, a constant strain rate was used for the definition since the TGO thickness is predefined to be 0.001 mm and its growth rate has little effect on the qualitative analysis of the stresses.

(2) $${\dot{h}}_{cr}=1.0\times 10^{-4}$$

A large number of experiments have proved that the growth of TGO at high temperatures is very complicated [39,40]. Due to the interface geometrical constraints and the influence of elemental diffusion, the growth strain at the peak is generally higher than the growth strain at the valley, and the growth strain between the peaks and valleys shows a continuous change trend. In this paper, to simulate the nonuniform growth of TGO, the growth strain is established as a function of the spatial coordinates, which shows a linear decrease from the peak to the valley, and the growth strain ${\dot{h}}_{\mathrm{peak}}=1.3\times 10^{-4}$ is set to be the peak growth strain, and the valley growth strain is set to be ${\dot{h}}_{\mathrm{valley}}=0.7\times 10^{-4}$.

### 2.5. Modeling Tool Used for Crack Initiation and Propagation

Failure of the coating is manifested by the coating spalling, which is generally caused by cracks between the TC/TGO. In this study, the cohesive element is applied to the TC/TGO interface to simulate the delamination of the interface, which is described by the law of traction separation, and the quadratic nominal stress criterion [41] is used in this paper.

(3) $${\left\{\frac{\langle \sigma_{n}\rangle }{\sigma_{n}^{0}}\right\}}^{2}+{\left\{\frac{\sigma_{s}}{\sigma_{s}^{0}}\right\}}^{2}+{\left\{\frac{\sigma_{t}}{\sigma_{t}^{0}}\right\}}^{2}=D$$

(4) $$\langle \sigma_{n}\rangle =\left\{ \begin{array}{l} \sigma_{n},\sigma_{n}\geq 0\\ 0,\sigma_{n}\leq 0 \end{array}\right.$$

where $\sigma_{n}$, $\sigma_{s}$ and $\sigma_{t}$ denote the nominal stresses when the deformation is completely perpendicular to the interface or completely along the first and second shear directions, respectively.$\sigma_{n}^{0}$, $\sigma_{s}^{0}$ and $\sigma_{t}^{0}$ are the peak values of interfacial tensile strength and shear strength, respectively. The BK criterion is used to determine the damage evolution [41].

(5) $$G_{n}{}^{c}+\left(G_{s}{}^{c}-G_{n}{}^{c}\right){\left\{\frac{G_{S}}{G_{T}}\right\}}^{\eta }=G^{c}$$

(6) $$G_{s}{}^{c}=G_{t}{}^{c}$$

(7) $$G_{S}=G_{s}+G_{t}$$

(8) $$G_{T}{=G}_{n}{+G}_{S}$$

where $G_{n}{}^{c}$, $G_{s}{}^{c}$, $G_{t}{}^{c}$ refer to the critical fracture energies when the fracture occurs purely in the normal or the first or the second shear direction, respectively, and *η* is a material parameter. In this model, these material parameters reported in Refs. [21,41,42] are used to describe the interfacial properties: critical interfacial strength $\sigma_{n}^{0}$ = $\sigma_{s}^{0}$= 100 MPa, critical fracture energy $G_{n}{}^{c}$ = $G_{s}{}^{c}$ = 0.02 mJ/mm^2^, η = 1.45. The interfacial modulus is calculated to be $K_{nn}$ = $K_{ss}$ = 0.50 × 10^7^ N/mm^3^.

(9) $$K_{nn}=10\sigma_{n}^{2}/G_{n}^{c}$$

(10) $$K_{ss}=10\sigma_{s}^{2}/G_{s}^{c}$$

## 3. Results and Discussions

### 3.1. Stress Distribution for the Uniform TGO

#### 3.1.1. Only the Thickening Growth

In this study, the shear stress *S*_12_ and normal stress *S*_22_ under different thermal loading cycles were investigated. For the cracking mode, the positive stress *S*_22_ tends to cause interface type I crack-dominated damage, while the shear stress *S*_12_ tends to cause interface type II crack-dominated damage. Based on the stress distribution and evolution at the interface, the cracking mode was investigated.

**Figure 6:**
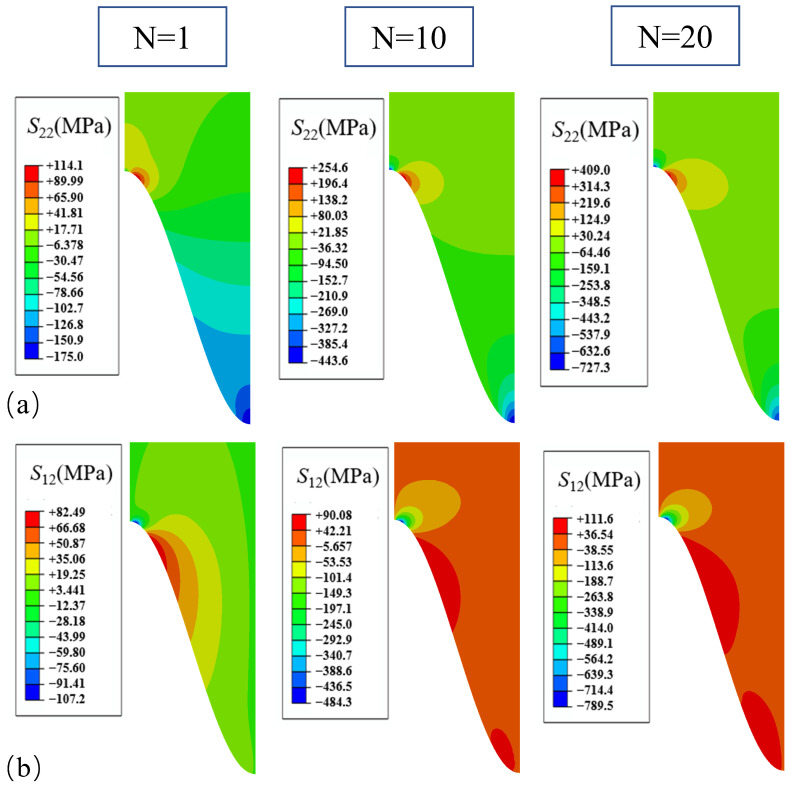
Stress distribution after different number of cycles with uniform growth in the thickening direction: (**a**) normal stress; and (**b**) shear stress.

The stress distributions, when only the growth in the thickening direction of the TGO is considered, for different numbers of thermal loading cycles are shown in Figure 6. It can be seen from Figure 6 that the normal stress *S*_22_ distribution characteristics remain unchanged after cooling down to room temperature with different numbers of thermal cycles. The peaks of normal stresses *S*_22_ all appear in the off-peak area, while the peak area gradually appears as a higher compressive stress region with the increased number of thermal cycles. The overall normal stress *S*_22_ distribution in the coating is compressive except for the off-peak area. The normal stress *S*_22_ in the off-peak area increased from 114 MPa to 409 MPa, while the normal stress *S*_22_ in the valley area increased from −175 MPa to −727 MPa. However, since positive values of the normal stress *S*_22_ represent tension and negative values represent compression, compression was not considered to cause damage in this study. The shear stress *S*_12_ showed a negative value in the off-peak area after one thermal cycle. As the thermal cycle proceeded, the value increased further from −107 MPa to −789 MPa. A region of positive values of shear stress *S*_12_ occurred at the interface below the off-peak area, and the coating as a whole is characterized by weak stresses except for this region. Since the shear stress *S*_12_ caused interface damage regardless of the positive or negative direction, it will be analyzed for absolute values.

**Figure 7:**
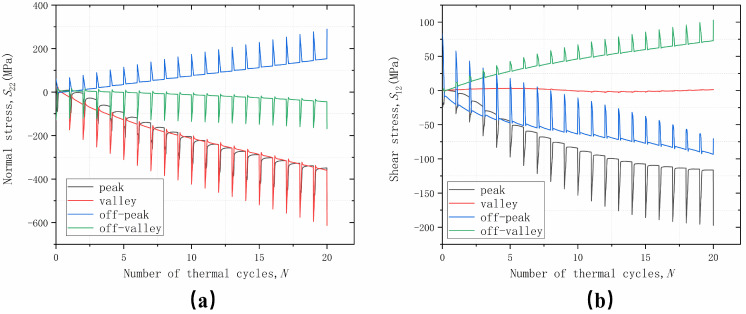
Stress variation with thermal cycles cycling at different positions considering uniform growth in the thickness direction: (**a**) normal stress; and (**b**) shear stress.

The trend of normal stress *S*_22_ and shear stress *S*_12_ at four positions with an increasing number of thermal loading cycles is shown in Figure 7. The cyclic variation of stresses reflects the effect of thermal mismatch stresses in the coating materials. The stress variation during the holding phase in each cycle reflects the influence of coating material properties and TGO growth. It can be seen that, due to the initial stress-free temperature set at 1000 °C, the stress levels are greatly increased after cooling to room temperature, which also indicates that damage is more likely to occur during the cooling phase, which is also consistent with the experimental results. While the overall trend change in stress is due to TGO growth, the change in the holding phase is a result of material plasticity, creep, and TGO growth stress competition. When the TGO is continuously thickening, the materials, such as BC, will yield or creep and cause the instability of the interface displacement, which leads to different stress states in different parts of the interface, and thus leads to different cracking behaviors.

From the change trend of normal stress *S*_22_, it can be seen that the stress in the off-peak area shows a linear increase with the increase in the number of cycles; the stress in the off-valley area only has a cyclic change caused by the temperature; and the stress in the peak and valley areas will converge to a stable value with the increase in the number of cycles. From the trend of shear stress *S*_12_, it can be seen that the stress in the valley area does not have obvious cyclic changes along with the temperature changes and shows no stress state. The stress in the off-peak area maintains an increasing trend with the increase in the number of cycles, but the magnitude of the stress change due to temperature change becomes smaller and smaller, and the stresses in the off-valley area and the peak area will converge to a stable value with the increase in the number of cycles. According to the distribution of normal stress *S*_22_ and shear stress *S*_12_, it can be inferred that a mixed failure of type I and type II cracks will occur in the off-peak area, and type II cracks will dominate the failure behavior in the peak and off-valley area, while damage does not easily occur in the valley area.

#### 3.1.2. Thickening and Lateral Growth

**Figure 8:**
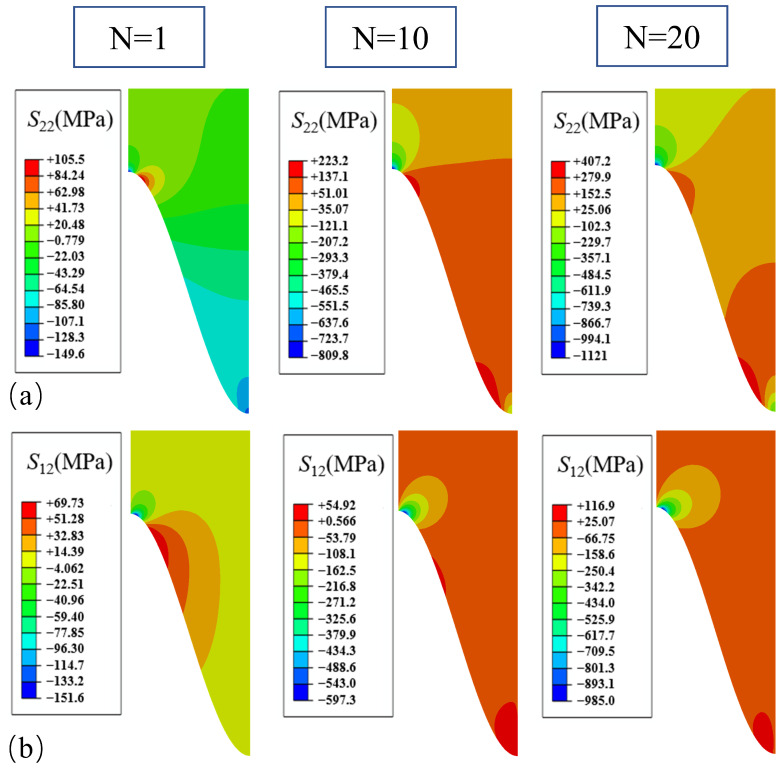
Stress distribution after different numbers of cycles considering the thickness and lateral uniform growth: (**a**) normal stress; and (**b**) shear stress.

The stress distribution with the number of cycles after considering the lateral growth is shown in Figure 8. After considering the lateral growth with a ratio of 0.1, it the results showed that that although the value for normal stress *S*_22_ did not change significantly, the stress distribution did change significantly. After one thermal cycle, the peak value appeared in the off-peak area, but along with the increase in the number of cycles, the peak site of the normal stress *S*_22_ changed and shifted from the off-peak area to the off-valley area. At the same time, the compressive stress in the valley area was improved to a certain extent, and the overall normal stress of the coating was in a tensile state. For shear stress *S*_12_, distribution does not have a large impact; the value of the off-peak area increased from 789 MPa to 985 MPa.

**Figure 9:**
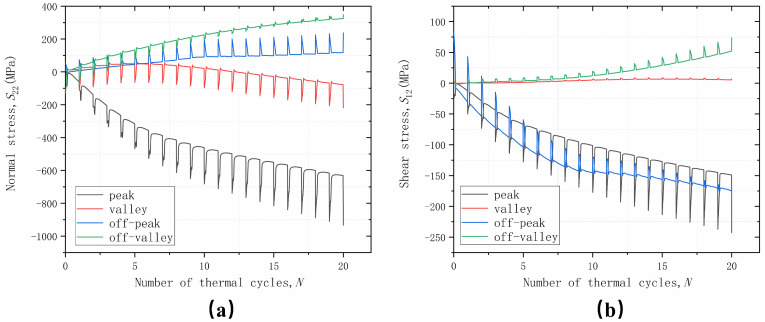
Considering the thickness and lateral uniform growth of different parts with thermal cycling stress variation curve:(**a**) normal stress; and (**b**) shear stress.

The trends of normal stress *S*_22_ and shear stress *S*_12_ with thermal cycling are shown in the Figure 9. From the stress trends at the four locations of *S*_22_, it can be seen that, under the influence of lateral growth, the off-valley area changes from the previous unstressed state to a tensile state and replaces the off-peak area to become the peak region of the normal stress *S*_22_. At the same time, the compressive stress in the valley area is released, while a larger compressive stress is introduced in the peak area. With the increase of thermal cycling, the valley area is gradually compressed, and the stress at the peak area gradually tends towards a stable value. The off-valley and the off-peak still have a growing trend, and the trend of the off-valley area is more significant than that of the off-peak area. From the trend of *S*_12_, the lateral growth causes the value of the off-peak area to further increase and reduces the shear stress in the off-valley area. The valley area remains stress-free, while the shear stress in the off-valley area will further increase with the number of cycles. This is because the lateral growth is considered to squeeze the off-valley area, leaving it in the tensile state after the interface is coordinated with the plasticity of the material by displacement. Based on the distribution of normal stresses *S*_22_ and shear stresses *S*_12_, it can be inferred that the mixed cracking behavior in the off-valley area remains. The failure behavior of the off-valley changes from type II cracking to a mixed failure of type I and type II cracking, with type II cracking dominating the failure behavior in the peak area, while damage is less likely to occur in the valley area.

### 3.2. Stress Distribution for Non-Uniform Growth

#### 3.2.1. Only the Thickening Growth

**Figure 10:**
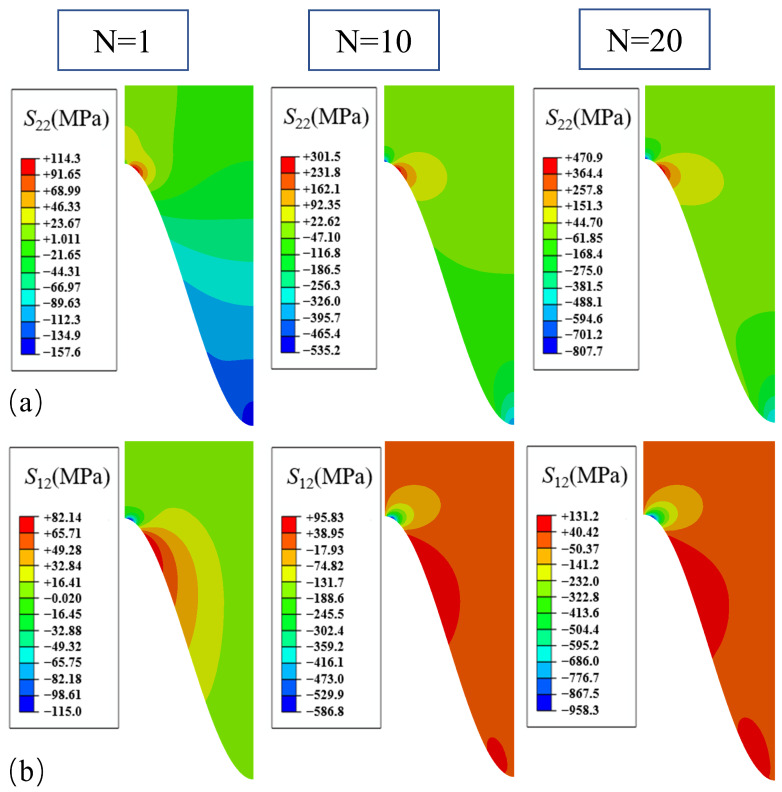
Stress distribution after considering the different numbers of cycles with non-uniform growth in the thickness direction: (**a**) normal stress; and (**b**) shear stress.

Considering only the non-uniform growth of TGO along the thickening direction, the growth strain is larger at the peak area and smaller in the valley area (compared to the uniform growth), during which the growth strain varies linearly, decreasing gradually from peak to valley. The variation of normal stress *S*_22_ and shear stress *S*_12_ with thermal cycling is shown in Figure 10. At the start of thermal cycling, the effect of non-uniform growth is not obvious due to the thin thickness of the TGO. With the gradual increase in thickness, the effect of non-uniform growth becomes gradually significant. After cooling to room temperature after 10 thermal cycles, although the distribution of normal stress *S*_22_ does not change significantly, the peak stress in the off-peak area increased by 20%, and still increased by 15% after 20 thermal cycles, which is due to the coordination of interfacial displacement as a result of material plasticity and the relaxation of stresses due to material creep. The shear stress *S*_12_ distribution is not affected by the non-uniform growth of the TGO and the values at the off-peak area are further increased by 25% after 10 thermal cycles and by 21% after 20 thermal cycles.

**Figure 11:**
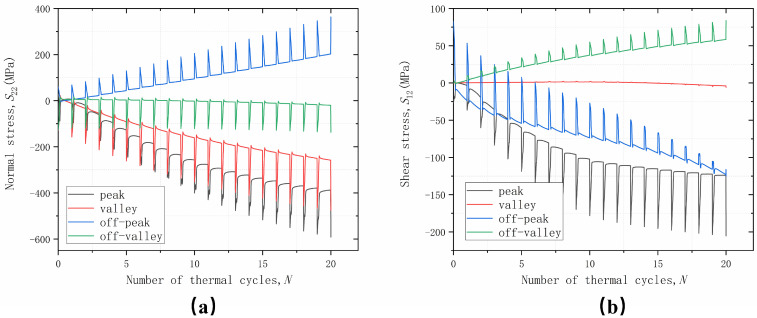
Stress variation curves with thermal cycling at different parts considering non-uniform growth in the thickness direction:(**a**) normal stress; and (**b**) shear stress.

The trends of normal stress *S*_22_ and shear stress *S*_12_ in the four areas with thermal cycling are shown in Figure 11. The trend of normal stress *S*_22_ with thermal cycling does not change significantly, and the compressive stress in the peak area is larger. The compressive stress in the valley area is reduced, which also indicates that the growth of TGO in the thickening direction has a greater effect on the normal stress *S*22. The shear stress *S*12 is also not changed due to the non-uniform growth of TGO, and the shear stress *S*12 in the off-peak area is further strengthened with the increase in the number of thermal cycles. This indicates that the non-uniform growth of TGO along the thickening direction will further deteriorate the stress level in the off-peak area, which will lead to the early generation of mixed cracks and failure in the off-peak area. There is no obvious difference between the cracking patterns in each area and those caused by the uniform growth of TGO.

#### 3.2.2. Thickening and Lateral Growth

**Figure 12:**
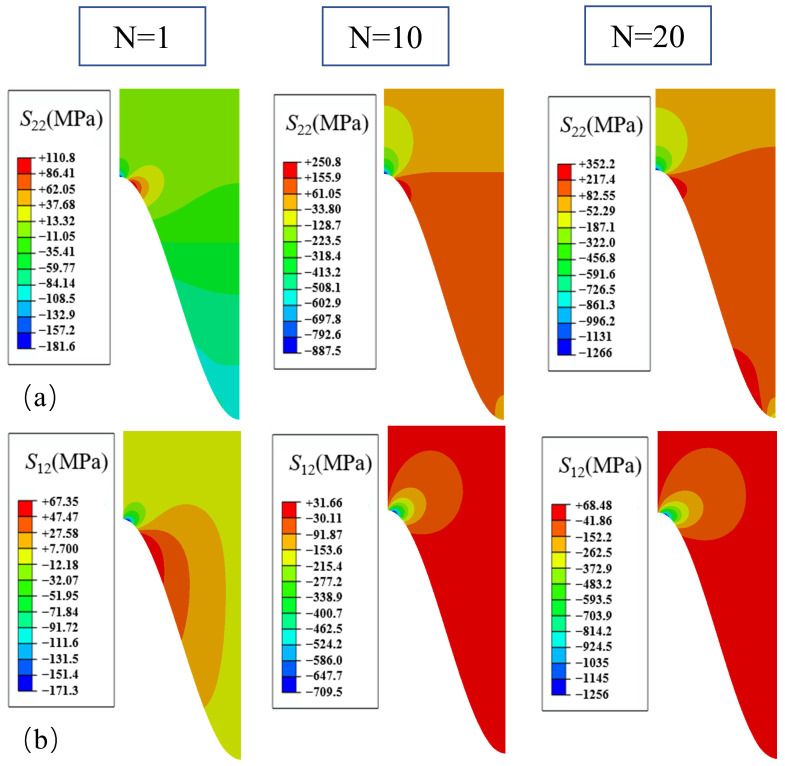
Stress distribution after a different number of cycles considering the thickness and lateral non-uniform growth:(**a**) normal stress; and (**b**) shear stress.

Based on the effect of non-uniform growth, the stress distributions of normal stress *S*_22_ and shear stress *S*_12_ after different thermal cycles are demonstrated in Figure 12. The figure shows that the consideration of lateral growth has a greater effect on the distribution of the overall normal stress *S*_22_ in the coating, which is transformed from a compressive to a tensile state, but releases the tensile stress in the peak area, which is reduced by 25% after 20 thermal cycles. The tensile stress in the off-peak area is reduced from 470 MPa to 352 MPa. Considering the lateral growth does not significantly affect the distribution of shear stress *S*_12_, but further increases the value of shear stress *S*_12_ in the off-peak area from 958 MPa to 1256 MPa after 20 thermal cycles. This is due to the lateral growth of the coating interface with different degrees of squeezing, resulting in the change in stresses in different parts of the interface.

**Figure 13:**
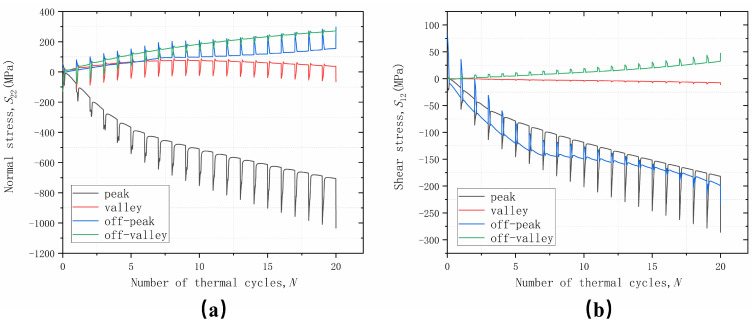
Considering the thickness and lateral non-uniform growth of different parts with thermal cycling stress variation curve:(**a**) normal stress; and (**b**) shear stress.

The trends of normal stress *S*_22_ and shear stress *S*_12_ in the four areas with thermal cycling are shown in Figure 13. The lateral growth causes the normal stress *S*_22_ in the off-valley area to increase and exceed the off-peak area to become the peak region of the normal stress *S*_22_, which causes the valley area to undergo a stress reversal from the previous compressive stress to the tensile state, and at the same time, reduces the value at the off-peak area. It is clear that the lateral growth reduces the shear stress in the off-valley area, raises the shear stress in the off-peak and peak areas, and also causes the peak area to continue to increase instead of showing a tendency to stabilize. The lateral growth increases the tensile separation stress in the off-valley area and decreases the tensile separation stress in the off-peak area. It greatly increases the shear separation stress in the off-peak as well as the peak area and decreases the shear stress in the off-valley area. The crack patterns in each area are not significantly different from those caused by the uniform growth of TGO.

### 3.3. Interfacial Cracking Behavior of TGO under Different Growth Modes

**Figure 14:**
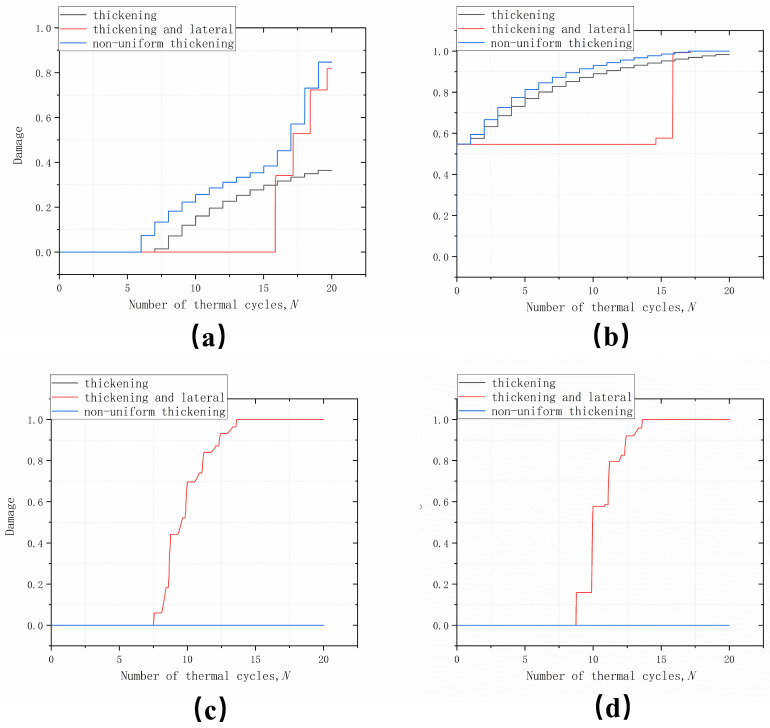
Damage evolution with the number of cycles in different areas in the modes: (**a**) peak; (**b**) off-peak; (**c**) off-valley; and (**d**) valley.

Four positions, peak, off-peak, off-valley and valley, were chosen to observe the change in damage with the number of cycles, as shown in Figure 12. In the peak area, as shown in Figure 14a below, the damage does not occur in the initial cycle. Rather, it occurs in subsequent cycles under the influence of the off-peak area. The analysis was performed using thickening growth as a baseline. The initial damage occurred after 7 thermal cycles for the thickening growth, while the thickening and lateral growth pattern delayed the damage to a greater extent, with the initial damage occurring after 16 thermal cycles. Considering the non-uniform growth pattern, the damage in the peak area was advanced, occurring after six thermal cycles. The trend of damage accumulation along the thickening growth was also more moderate, and the thickening and lateral growth pattern with non-uniform growth accumulated substantially after 16 thermal cycles. This was due to the fact that cracking had already occurred off-peak after 16 cycles.

For the off-peak area, as shown in Figure 14b below, the damage occurred at the initial progression of the cycle due to the fact that the area has the harshest stress conditions, including normal and shear stresses. However, the different growth modes had very different effects on the damage accumulation in this area. Considering the thickening growth mode as a basis, the thickening and lateral growth greatly retarded the number of thermal cycles of damage accumulation, which were delayed from 1 thermal cycle to 14 thermal cycles before damage accumulation began to occur. Non-uniform growth, on the other hand, accelerated damage accumulation in the area to some extent. Damage accumulation trends were more moderate for both thickening and non-uniform growth, except that non-uniform accumulation was greater. For the thickening and lateral growth pattern, damage accumulation increased abruptly after 16 thermal cycles.

The variation of damage with the number of thermal cycles in the off-valley and valley areas is shown in Figure 14c,d below. Apart from thickening and lateral growth, no damage occurred during thickening and non-uniform growth. The off-valley damage started to occur after seven thermal cycles, followed by valley damage at eight thermal cycles. The trend was similar for both areas, with damage accumulating more rapidly from the initial damage and reaching “1” after 13 thermal cycles, i.e., interfacial cracking occurred. This was also the most significant feature of thickening and lateral growth, which led to a significant change in the cracking site during the thermal cycling process, shifting from the off-peak area to the off-valley and the valley area. This demonstrates the strongest evidence for the presence of thickening and lateral growth in the test results in the presence of off-valley and valley area cracks. For the off-valley and valley areas, no damage occurred under conditions of growth along the thickening direction as well as non-uniform growth. So in Figure 14c,d the non-uniform growth covers the damage accumulation curves growing along the thickening direction.

**Figure 15:**
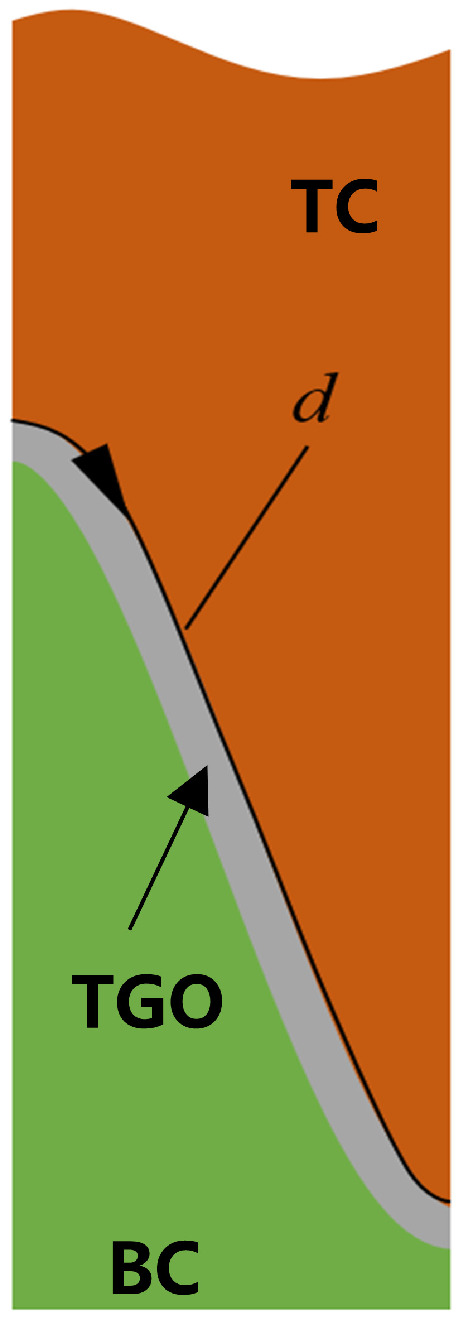
Schematic diagram of the interface path.

**Figure 16:**
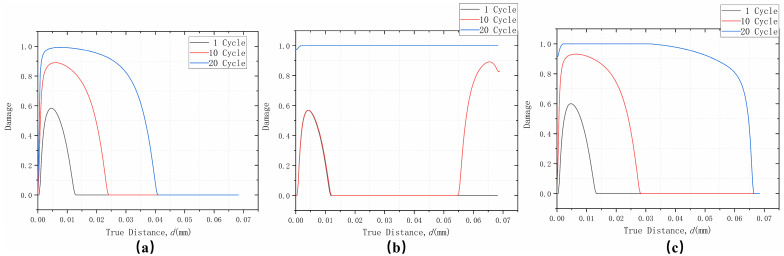
Damage distribution after different numbers of cycles at the interface considering: (**a**) uniform thickening growth; (**b**) thickening and lateral growth; and (**c**) non-uniform thickening growth.

The interface path shown in Figure 15 was selected with the direction from left to right; d represents the distance from the left boundary. Figure 16 shows the interface damage on this path after 1, 10, and 20 thermal cycles. Different TGO growth modes will have a large impact on the occurrence of interfacial damage expansion. From the overall trend, it was found that the thickening and non-uniform growth were similar, with the damage occurring off-peak area and expanding to both sides. The only difference in the results is that the interfacial damage expanded faster in the non-uniform growth. Thickening and lateral growth, on the other hand, altered the occurrence of interfacial crack expansion, and although damage initially occurred in the off-peak area, it did not continue to accumulate with thermal cycling. Instead, off-valley area damage occurred, and cracking occurred throughout the interface after 20 cycles. That is, the thickening and lateral growth changed the interface crack expansion to a large extent, expanding from the previous off-peak area to both sides. This changed to off-valley expansion at both sides, and then changed to an off-valley area and off-peak area expansion to both sides afterwards. At the same time, it also greatly shortened the time for interfacial cracks to appear, leading to premature coating failure. The destructive nature of the thickening and lateral growth pattern was therefore characterized.

**Figure 17:**
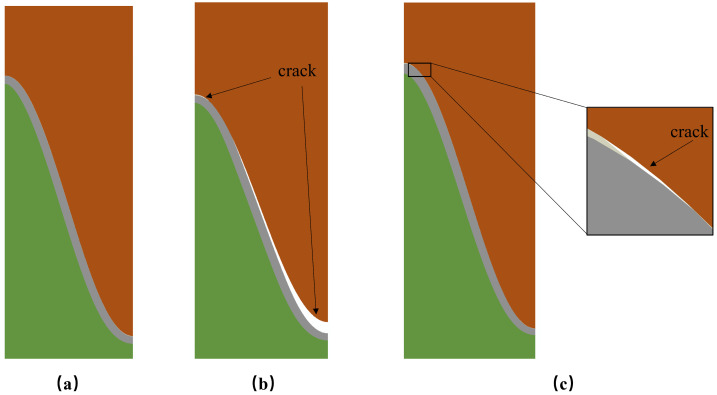
Interface cracking behavior after 20 thermal cycles considering: (**a**) uniform thickening growth; (**b**) thickening and lateral growth; and (**c**) non-uniform thickening growth.

The interfacial cracking behavior due to different TGO growth modes after 20 thermal cycles is demonstrated in Figure 17 below. When only the growth in the TGO thickening direction was considered, the TC/TGO interface was damaged to a certain extent, but no cracks were found (the cracking behavior occurred when the damage reached 1). When the lateral growth of TGO was considered, substantial cracking occurred at the interface, and the degree of cracking in the valley area was much larger than that in other areas of the interface. When the non-uniform growth of TGO was considered, it was found that the cracking had occurred in the off-peak area. The macroscopic cracking behavior demonstrates the effect of different TGO growth modes on the interface. Non-uniform growth has a promoting effect on the interface cracking, while lateral growth will lead to substantial cracking of the interface.

## 4. Conclusions

In this paper, a systematic analysis of coating stress distribution and interfacial cracking based on different growth modes of TGO was carried out. The distribution and evolution of normal and shear stresses were first investigated, and the interfacial damage was then simulated by cohesive elements. The main conclusions are as follows:

(1) When only uniform growth in the direction of TGO thickening is considered, the worst stress conditions are found in the off-peak region. When lateral growth is also considered, tensile separation stresses to the off-valley region will increase. The shear separation stress *S*_12_ in the off-peak region will further increase;
(2) When only non-uniform growth in the direction of TGO thickening is considered, the harshness of the stress in the off-peak region is further exacerbated. Lateral growth will increase the tensile separation stress in the off-valley region and decrease the tensile separation stress in the off-peak region. In addition, it greatly increases the shear separation stresses in the off-peak and peak regions and decreases the shear stresses in the off-valley region;
(3) Lateral growth has a greater influence on the interfacial damage and cracking behavior, which will lead to a change in the crack extension mode from the off-valley area as the crack initiation point. The non-uniformity accelerates the occurrence of failure behavior in the off-peak and peak areas, and also leads to destructive behavior in the off-valley area.

## Figures and Tables

**Table 1:** Thermo-physical parameters for all materials [33].

Material	Temperature/ °C	Elastic Modulus/ GPa	*μ*	Density/ (g·cm^−3^)	Thermal Expansion Coeff./ (10^−6^·K^−1^)	Thermal Conductivity/ (W·m^−1^·K^−1^)	Specific Heat/ (J·kg^−1^·K^−1^)
TC	25	17.5	0.20	5.650	9.68	1.05	483
	400	-	0.20	5.650	9.70	1.05	483
	800	-	0.20	5.650	9.88	1.05	483
	1000	12.4	0.20	5.650	10.34	1.05	483
TGO	25	380	0.27	3.978	5.10	25.2	857
	400	-	0.27	3.978	-	25.2	857
	800	338	0.27	3.978	-	25.2	857
	1000	312	0.27	3.978	9.80	25.2	857
BC	25	183	0.30	7.320	-	4.6	501
	400	152	0.30	7.320	12.50	6.4	593
	800	109	0.30	7.320	14.30	10.2	781
	1000	-	0.30	7.320	16.00	16.1	764
DZ411	25	129.9	0.30	8.344	-	8.6	469
	400	118	0.30	8.344	12.90	15.5	501
	800	101	0.30	8.344	14.50	21.1	547
	1000	86	0.30	8.344	15.60	23.1	575

**Table 2:** Yield strength of all materials [34,35].

Layers	Temperature/°C	Plastic Strain	*σ*_y_/MPa
TGO	20	0	10,000
	900	0	10,000
	1000	0	1000
BC	20	0	1000
	300	0	1000
	750	0	100
	1000	0	100
DZ411	20	0	1280
	650	0	1255
	700	0	1185
	800	0	955
	900	0	655
	980	0	595
	1000	0	356

**Table 3:** Creep parameters of different materials [36].

Layers	B (s^−1^·MPa^−n^)	n	T (°C)
TC	1.8 × 10^−9^	1	1000
TGO	7.3 × 10^−9^	1	1000
BC	6.54 × 10^−19^	4.57	≤600
	2.20 × 10^−12^	2.99	700
	1.84 × 10^−7^	1.55	800
	2.15 × 10^−8^	2.45	≥850

## Data Availability

The data are not publicly available because they also form part of an ongoing study.
